# Subjective stress reactivity in psoriasis – a cross sectional study of associated psychological traits

**DOI:** 10.1186/s12895-015-0026-x

**Published:** 2015-05-02

**Authors:** Charlotta Remröd, Karin Sjöström, Åke Svensson

**Affiliations:** Department of Dermatology and Venereology, University of Lund, Hudkliniken, Skåne University Hospital, Jan Waldenströmsg. 16, Malmö, 205 02 Sweden; Psychiatric consultant at the Department of Dermatology and Venereology, Hudkliniken, Skåne University Hospital, Jan Waldenströmsg. 16, Malmö, 205 02 Sweden

**Keywords:** Plaque psoriasis, Psychology, Stress, Anxiety, Depression, Personality assessment

## Abstract

**Background:**

Stress or psychological distress is often described as a causative or maintaining factor in psoriasis. Psychological traits may influence the appraisal, interpretation and coping ability regarding stressful situations. Detailed investigations of psychological traits in relation to stress reactivity in psoriasis are rare. The aim of this study was to examine whether patients with psoriasis who report an association between psychological distress and exacerbation, “stress reactors” (SRs), differ psychologically from those with no stress reactivity “non-stress reactors” (NSRs).

**Methods:**

This cross-sectional study was conducted among 101 consecutively recruited outpatients with plaque psoriasis. A psychosocial interview was performed including questions concerning stress reactivity in relation to onset and exacerbation. Three validated self-rating scales were used: Spielberger State-Trait Anxiety Inventory (STAI, Form-Y), Beck Depression Inventory (BDI-II) and Swedish Universities Scales of Personality (SSP). Independent samples t-tests, Chi-square tests and one-way ANOVA analyses were used for group comparisons when appropriate. A logistic regression model was designed with SR as the dependent variable.

**Results:**

Sixty-four patients (63%) reported a subjective association between disease exacerbation and stress (SRs). Patients defined as SRs reported significantly higher mean scores regarding state and trait anxiety, depression, and also five SSP scale personality traits, i.e. somatic trait anxiety, psychic trait anxiety, stress susceptibility, lack of assertiveness and mistrust, compared with NSRs. In multivariate analysis, SSP-stress susceptibility was the strongest explanatory variable for SR, i.e. OR (95% CI) = 1.13 (1.02 – 1.24), p *= 0.018.*

**Conclusion:**

According to our results, patients who perceive stress as a causal factor in their psoriasis might have a more vulnerable psychological constitution. This finding suggests important opportunities for clinicians to identify patients who may benefit from additional psychological exploration and support.

## Background

Psoriasis is one of the most common immune-mediated skin diseases and is known to have a systemic inflammatory involvement [1]. The estimated prevalence of psoriasis is 1.5 – 3% in Scandinavia and Northern Europe [2,3]. A genetic–environmental interaction seems to offer a plausible aetiological explanation of psoriasis [1], and psychological distress has often been suggested as an important trigger [4,5]. Only a few prospective studies of stress and psoriasis exist [4,6,7], and the associations and mechanisms involved remains unclear. Despite the lack of strong aetiological evidence for the association between psychological distress and psoriasis, between 37% and 71% of patients report psychological distress as one of the major causative agents for onset, exacerbation and maintenance of their psoriasis [8-15]. These patients may be defined as stress reactors. Psychological distress has also been found to reduce efficacy of treatment in psoriasis [16], and improvement of clinical parameters as a result of psychological interventions adds further evidence for the association between psychological distress and psoriasis [17,18].

Nevertheless, research has shown that focusing solely on stressors from the environment is too simplistic [19,20]. Almost no single life event would be regarded as a stressor by all individuals with such exposure, because individual responses are highly influenced by perceptions and interpretation of events. Genetic, personal, emotional and social factors will determine whether an individual can tolerate and overcome the effects of stressors [19,20]. Consequently, psychological traits are of significant importance in stress theory and research. Personality influences both the exposure to, and appraisal of stressful situations, and also the individual’s interpretation of stressors and coping ability [19]. Some psychological traits can possibly predict increased emotional and physiological reactivity under stressful conditions [19-22].

Although stress has been recognised as an important factor within the field of psoriasis research, detailed investigations of psychological traits and clinical characteristics in relation to perceived stress-reactivity are rare. One of the few larger studies to elucidate this subject was conducted by Zachariae et al., [8] who found an association between subjective stress-reactivity and indicators of psychological vulnerability. Some smaller studies have reported subjective stress-reactivity to be associated with poorer levels of psychosocial well-being [13], pathological worrying [7,23], difficulties with assertion of anger, and dependency upon approval [9]. Devrimci-Ozguven et al. [14] showed conflicting results with no association between stress-reactivity and psychological morbidity. To the best of our knowledge, no previous study has as yet used a larger structured personality inventory in an investigation of stress reactivity in psoriasis.

The aim of this study was to examine the subjective influence of stress on psoriasis onset and exacerbations. Furthermore, we wanted to compare persons characterised as “stress reactors” (SRs) and “non-stress reactors” (NSRs) with respect to psychological variables, clinical- and socio-demographic factors and psoriasis-related distress. We hypothesise that stress-reactors have a more psychologically vulnerable constitution, as compared with non-stress reactors. By psychological vulnerability, we refer to an individual’s inability to withstand the effects of a potentially stressful environment, due to psychological sensitivity and lack of adequate coping mechanisms.

## Method

### Subjects

All subjects were recruited consecutively from planned visits at the out-patient clinic of the Department of Dermatology and Venereology at the Skåne University Hospital in Malmö, Sweden. Inclusion criteria were: plaque psoriasis diagnosed by dermatologist, men and women aged 18 – 65 years, good command of the Swedish language, and no serious mental or cognitive disturbances. A total of 109 patients were approached during early autumn 2008 (53%) and autumn 2009 (47%). Of them, 102 agreed to participate (94%) and gave their oral and written informed consent. One patient dropped out of the study, due to personal considerations. All of the 101 (93%) remaining patients were unpaid volunteers. A comparison of the two cohorts from 2008 and 2009 showed no statistically significant differences regarding any socio-demographic and clinical variables. The subjects were accordingly regarded as one cohort in statistical analyses.

No statistically significant differences were found between men and women regarding socio-demographic and clinical variables, psychosocial-, psychological-, and psoriasis-related variables.

### Methods

A psychosocial semi-structured 25-item interview was conducted in a quiet room at the out-patient clinic. All subjects were interviewed by the same researcher (CR). The interview was designed by two of the authors (KS and CR), with the purpose of assessing (i) socio-demographic variables, (ii) social situation and close relationships, and (iii) psoriasis-related distress. Answers were rated on a 5-point Likert scale. Regarding (ii) social situation and close relationships, patients were asked about satisfaction with living conditions, working conditions, private economy and satisfaction with relationships with mother, father, partner, children, friends and colleagues. Answers were dichotomised as “satisfied” (1–3) and “not satisfied” (4–5). Regarding (iii) psoriasis-related distress, patients were asked about their psoriasis impact on daily life and on sexual relations. Answers were dichotomised as “low impact” (1–3) and “high impact” (4–5).

At the end of the interview, patients were asked: (A) “Do you relate the onset of your psoriasis to a particular stressful life situation?” (Answers were given as “yes”, “no”, “don’t know”), and (B) “Do you experience that your psoriasis is aggravated during times of stress?” (Answers were given as “yes”, “no”, “sometimes” or “don’t know”). For question (B), two groups were created for group comparisons, i.e. “stress reactors” (SRs) = (yes) and “non-stress reactors” (NSRs) = (no).

All patients were asked to rate their general degree of pruritus on a Visual Analogue Scale (VAS). The scale consisted of a 10 cm straight line without numbers or sections. The left end was labelled “no pruritus”, and the right end was labelled “severe pruritus”.

After the interview, each patient was given privacy to complete three psychometric self-rating scales in a quiet room, with the researcher readily available for questions in a room nearby.

### Spielberger state-trait anxiety inventory (STAI)

The STAI (Form-Y) is a well-established self-rating scale with high stability and validity, often used in clinical research [24]. The first 20 statements assess state anxiety, i.e. anxiety at a particular moment or at a chosen period of time. (The subjects were asked to rate their state anxiety during the last week). The subsequent 20 statements assess trait anxiety, i.e. the relatively stable anxiety proneness. Answers are given on a 4-point Likert scale, and scores on the state and trait scales, respectively, range from 20 to 80 points. In large normative samples of working adults and college students, the mean values of state and trait anxiety for men range from 35.7 to 36.5 and 34.9 to 38.3, respectively, and for women from 35.2 to 38.8 and 34.8 to 40.4, respectively [24].

### Beck depression inventory (BDI-II)

The Beck Depression Inventory Second Edition (BDI-II) is one of the most widely used self-report measures of depression in both research and clinical practice, with high validity and good psychometric properties [25]. The questionnaire consists of 21 items, and answers are rated on a four-point scale (0 = low, 3 = high). The total score ranges from 0 to 63. For persons who have been clinically investigated for depression, scores from 0–13 represent minimal depressive symptoms, scores of 14–19 indicate mild, scores of 20–28 indicate moderate, and scores of 29–63 indicate severe depressive symptoms [25]. Question number 16 evaluates sleep disturbances. Since sleep disturbances may be associated both with depression and stress reactivity, this variable was extracted, dichotomised and used in statistical analyses.

### Swedish universities scales of personality (SSP)

The SSP is a thorough revision of the older Karolinska Scales of Personality (KSP). In contrast to many other personality inventories, SSP does not intend to measure “the entire personality”, but has been developed to identify stable traits of psychological vulnerability and psychopathology. Psychological vulnerability is believed to predispose the individual to psychological problems [26]. The questionnaire comprises 91 items with a 4-point Likert response scale. The items are sorted into 13 subscales, each designed to measure one personality trait: (1) Somatic Trait anxiety, (2) Psychic Trait Anxiety, (3) Stress Susceptibility, (4) Lack of Assertiveness, (5) Impulsiveness, (6) Adventure Seeking, ( 7) Detachment, (8) Social Desirability, (9) Embitterment, (10) Trait Irritability, (11) Mistrust, (12) Verbal Trait Aggression, and (13) Physical Trait Aggression [27]. The SSP has been standardised in a large representative Swedish national sample, and the internal consistency with regard to Cronbach’s alpha coefficient ranged from 0.59 to 0.84 in a normative sample [26]. The subscales are transformed into *T* scores according to the SSP computer algorithm. *T* scores (mean 50, SD 10) are standardised with regard to age and sex on the basis of a normal control group. Values of 10 points above or below 50 in each SSP scale indicate a difference from the standard population by 1.0 SD [27].

### Psoriasis area and severity index (PASI)

Clinical assessment of PASI was conducted on 48 patients recruited during autumn 2009. The PASI scoring system is currently the best evaluated and the most widely used objective method to evaluate clinical severity of psoriasis [28]. The PASI combines the assessment of the area affected and the severity of lesions into a single score ranging from 0 (no disease) to 72 (severe disease). Severity has been categorised as follows: PASI < 7 = mild plaque psoriasis, PASI 7–12 = moderate plaque psoriasis, PASI > 12 = severe plaque psoriasis [29].

### Statistical analysis

Independent samples t-tests, Chi-square tests and one-way ANOVA analyses were used for group comparisons when appropriate. Post hoc multiple comparisons were performed, using Tukey’s test to identify pairwise significant differences. A logistic regression model was designed with SR as the dependent variable. All psychometric variables with a significant difference between SR and NSR in group comparisons were included and analysed in the model. The psychometric variables were first analysed separately, then adjusted for potential covariates, and finally with all psychometric variables and covariates included in the same model. Covariates used in the final model were age, gender, psoriasis impact on daily life, and age at debut of psoriasis. Age at debut was included since an association between this variable and psychological vulnerability has previously been found [30]. Other potential covariates that did not reach significance in the first adjustment were excluded from the final model, and they were: psychosocial variables sleep disturbances, alcohol consumption, PASI, pruritus and BMI. Two-tailed *p*-values < 0.05 were considered to be statistically significant. Statistical analyses were carried out using the Statistical Package for the Social Sciences, version 21.0 (SPSS™, Chicago, IL, USA).

The Ethics Committee of the Medical Faculty, University of Lund approved the study.

## Results

Socio-demographic and clinical characteristics of the sample are given in Table 1.

**Table 1:** **Socio-demographic and clinical characteristics** ***(N = 101)***

**Characteristics**	
Gender, *n* (%)	
Male	56 (55)
Female	45 (45)
Age (years)	
Mean (SD), Mdn (range)	43.5 (13.8), 45 (18–65)
Age at onset of disease (years)	
Mean (SD), Mdn (range)	24.7 (14.5), 20 (0–61)
Duration of disease (years)	
Mean (SD), Mdn (range)	18.8 (12.7), 18 (1–56)
BMI	
Mean (SD), Mdn (range)	26.2 (4.5), 26.3 (17.6-38.5)
PASI (*n* = 48)	
Mean (SD), Mdn (range)	5.4 (4.3), 4.2 (0–21.7)
Mild psoriasis, *n* (%)	37 (77)
Moderate psoriasis, *n* (%)	8 (17)
Severe psoriasis, *n* (%)	3 (6)
Marital status, *n* (%)	
Single	18 (18)
Partner/cohabiting	35 (35)
Married	39 (38)
Divorced or widow/widower	9 (9)
Educational level, *n* (%)	
1-9 years	11 (11)
10-12 years	37 (37)
>12 years	53 (52)
Employment, *n* (%)	
Full time	66 (65)
Part time	13 (13)
Unemployed / retired	22 (22)
Tobacco consumption, *n* (%)	
Smokers	32 (32)
Nonsmokers	69 (68)
Alcohol consumption (gram/week)	
Mean (SD), Mdn (range)	45.9 (59.8), 30 (0–360)
Risk consumption:^a^	
Men, *n* (%)	3 (3)
Women, *n* (%)	2 (2)

BMI, Body Mass Index; PASI, Psoriasis Area and Severity Index.

^a^Alcohol risk consumption (standard drinks/week); men > 14, women > 9.

### Psychosocial interview

Most patients were satisfied with living conditions (93%), working conditions (98% of n = 90), private economy (89%) and also with relationships with mother (92% of n = 99), father (86% of n = 94), partner (91% of n = 77), own children (100% of n = 66), friends (94%) and colleagues (98% of n = 80). Forty-nine patients (48%) reported that their psoriasis had a high impact on their daily life, and 27 patients (27%) reported that their psoriasis had a high impact on sexual relations.

### Stress and exacerbation

Sixty-four patients (63%) were defined as “stress reactors” (SRs), 26 patients (26%) “non- stress reactors” (NSRs), seven patients (7%) answered “don’t know” and four patients (4%) “sometimes”. Statistically significant differences between SRs and NSRs were found regarding mean scores of state, and trait anxiety, BDI-II and five personality traits on the SSP scale, i.e. somatic trait anxiety, psychic trait anxiety, stress susceptibility, lack of assertiveness and mistrust. Results are presented in Table 2 together with descriptive mean scores for the total sample and all four groups of subjective stress-reactivity. No statistically significant differences between SRs and NSRs were found regarding all socio-demographic and clinical variables shown in Table 1, psychosocial variables, psoriasis-related distress or sleep disturbances.

**Table 2:** **Results from the psychometric scales** ***Mean scores from the total sample, for the different groups of subjective stress reactivity and group comparisons of SRs vs. NSRs***

	**Total**	**“Yes” (SR)**	**”No” (NSR)**	**“Don’t know”**	**“Sometimes”**	**SR vs. NSR Significance of difference,** ***p***
	**Mean (SD)**	**Mean (SD)**	**Mean (SD)**	**Mean (SD)**	**Mean (SD)**	
	**(** ***N*** **= 101)**	**(** ***n*** **= 64)**	**(** ***n*** **= 26)**	**(** ***n*** **= 7)**	**(** ***n*** **= 4)**	
**STAI**						
State anxiety	38.0 (12.2)	40.5 (12.5)	29.9 (8.0)	44.0 (10.1)	40.8 (10.9)	<0.0001
Trait anxiety	36.5 (11.9)	38.3 (12.1)	28.0 (6.4)	43.1 (13.3)	35.0 (12.0)	<0.0001
**BDI-II** Depression	8.4 (8.1)	10.0 (8.5)	3.4 (3.6)	13.1 (10.4)	7.0 (4.1)	<0.0001
**SSP**						
Somatic Trait Anxiety	51.2 (10.8)	53.2 (10.4)	45.1 (9.8)	58.1 (10.8)	47.1 (4.9)	0.001
Psychic Trait Anxiety	47.5 (9.9)	49.2 (10.6)	42.1 (6.3)	53.3 (6.7)	46.6 (12.6)	<0.0001
Stress Susceptibility	50.7 (10.9)	53.5 (10.9)	42.1 (7.7)	55.4 (7.1)	53.9 (7.8)	<0.0001
Lack of Assertiveness	47.0 (9.9)	48.9 (10.6)	42.1 (6.1)	49.9 (10.8)	43.5 (4.5)	<0.0001
Impulsiveness	50.8 (9.9)	51.3 (10.0)	50.0 (10.9)	52.3 (6.7)	46.6 (9.1)	n.s.
Adventure seeking	49.7 (9.3)	49.5 (9.9)	49.5 (8.5)	51.6 (8.6)	52.4 (8.5)	n.s.
Detachment	47.0 (10.2)	47.2 (10.3)	44.6 (9.6)	53.5 (7.7)	47.5 (15.7)	n.s
Social Desirability	51.2 (9.7)	51.6 (10.5)	51.5 (9.9)	47.4 (7.6)	49.5 (5.4)	n.s
Embitterment	50.6 (10.1)	51.9 (10.0)	47.7 (8.3)	51.9 (8.2)	45.3 (2.6)	n.s.
Trait Irritability	49.3 (10.4)	49.9 (11.2)	46.3 (9.1)	55.0 (9.4)	48.7 (2.0)	n.s.
Mistrust	48.9 (11.6)	49.8 (11.7)	43.7 (10.6)	59.1 (8.2)	50.3 (6.1)	0.024
Verbal Trait Aggression	49.5 (10.7)	48.9 (11.1)	50.3 (10.4)	53.5 (10.7)	48.2 (7.9)	n.s.
Physical Trait Aggression	48.0 (10.2)	47.8 (9.6)	48.2 (11.0)	54.2 (12.7)	39.3 (5.9)	n.s.

SR: stress reactors; NSR: non-stress reactors; STAI: State- and Trait anxiety Inventory; BDI-II: Beck Depression Inventory-II; SSP: Swedish Universities Scales of Personality.

In multivariate logistic regression analysis, the psychometric variables tested were all significant explanatory variables for SR when analysed as single variables. When these analyses were controlled for potential covariates, all psychometric variables remained significant, except SSP-mistrust. When analysing all psychometric variables with covariates in the same model, SSP-stress susceptibility was the only significant explanatory variable for SR. Due to multi-collinearity between the psychometric variables, the other psychometric variables did not remain significant. Odds-ratios (95% CI) and *p-*values are presented in Table 3.

**Table 3:** **Results from the logistic regression analyses with “stress reactor” as dependent variable** ***(N =90)***

**Psychometric variables**	**Analysed as single variables**		**Analysed as single variables with covariates** ^**a**^		**All psychometric variables with covariates** ^**a**^ **in the same model**	
	**OR (95% CI)**	***p***	**OR (95% CI)**	***p***	**OR (95% CI)**	***p***
**STAI** Trait anxiety	1.14 (1.06 – 1.22)	<0.0001	1.14 (1.06 – 1.23)	<0.0001	1.10 (0.97 – 1.24)	0.136
**BDI-II** Depression	1.20 (1.07 – 1.34)	0.001	1.22 (1.08 – 1.38)	0.002	1.11 (0.92 – 1.35)	0.269
**SSP** Somatic trait anxiety	1.08 (1.03 – 1.14)	0.002	1.08 (1.02 – 1.14)	0.005	1.05 (0.96 – 1.14)	0.322
Psychic trait anxiety	1.09 (1.03 – 1.16)	0.004	1.09 (1.02 – 1.16)	0.007	0.85 (0.71 – 1.02)	0.083
Stress susceptibility	1.14 (1.07 – 1.23)	<0.0001	1.15 (1.07 – 1.24)	<0.0001	1.13 (1.02 – 1.24)	0.018
Lack of assertiveness	1.09 (1.03 – 1.15)	0.005	1.09 (1.02 – 1.16)	0.008	1.08 (0.98 – 1.19)	0.104
Mistrust	1.06 (1.01 – 1.11)	0.028	1.05 (0.99 – 1.10)	0.061	0.98 (0.91 – 1.06)	0.657

STAI: Spielberger State- and Trait anxiety Inventory, BDI-II: Beck Depression Inventory-II, SSP: Swedish Universities Scales of Personality.

^a^Covariates: age, gender, psoriasis impact on daily life, age at debut of psoriasis.

### SSP-stress susceptibility

Scores of SSP-stress susceptibility were positively correlated with scores of state and trait anxiety (r = 0.61 and r = 0.73 respectively, *p* < 0.0001), scores of BDI-II (r = 0.61, *p* < 0.0001), SSP-somatic trait anxiety (r = 0.60, *p* < 0.0001), SSP-psychic trait anxiety (r = 0.75, *p* < 0.0001), SSP-lack of assertiveness (r = 0.47, *p* < 0.0001) and SSP-mistrust (r = 0.50, *p* < 0.0001). No significant correlation or mean differences were found between SSP-stress susceptibility and of the socio-demographic and clinical variables shown in Table 1, psychosocial variables or sleep disturbances.

### Stress and onset of psoriasis

Fifty patients (49%) reported an experience of disease onset during a stressful life situation. Thirty-seven patients (37%) did not, and 14 patients (14%) answered “don’t know”. Patients with onset related to stress had significantly higher mean age at onset compared with those who answered “no” (29.2 years vs 21.8 years, *p* = 0.040) and those who answered “don’t know” (15.9 years, *p* = 0.005). Compared with those who answered “no”, patients with onset related to stress had significantly higher mean scores of state anxiety (40.1 vs. 34.1, *p* = 0.045), trait anxiety (38.9 vs. 32.2, *p* = 0.023), BDI-II (10.0 vs. 5.8, *p* = 0.043) and SSP-psychic trait anxiety (49.3 vs. 43.9 *p* = 0.016). No statistically significant differences between the three groups were found regarding the other socio-demographic and clinical variables shown in Table 1, other SSP-traits, psychosocial variables, psoriasis-related distress or sleep disturbances.

## Discussion

In our study, more than half of the patients reported stress as a causative agent for exacerbation of their psoriasis. Subjective stress reactivity (SR) was associated with both higher scores of depression, anxiety, and also five personality traits on the SSP scale, i.e. stress-susceptibility, somatic anxiety, psychic anxiety, lack of assertiveness and mistrust. SSP-stress susceptibility showed the strongest association with SR in multivariate regression analysis and seems to be the most relevant personality trait in this study.

Individuals with high scores of SSP-stress susceptibility more often state that they “get tired and hurried too easily”, “can not handle being interrupted when working with something”, “in order to get something done have to spend more energy than most others do”, “have difficulties to concentrate on what I’m doing if the environment is distracting”, “easily feel pressure when told to speed up work”, “feel insecure when facing new tasks”, “think I have less energy than most people I know” [27]. SSP-stress susceptibility showed medium to high correlations with scores of anxiety, depression, SSP-somatic- and -psychic anxiety, SSP-lack of assertiveness and SSP-mistrust. Hence, these individuals are likely to experience and encounter more stress in their daily life compared with less psychologically vulnerable individuals, which in turn may increase stress- related immune dysregulation [31].

To the best of our knowledge, this study is one of the few to thoroughly investigate psychological traits in relation to subjective stress-reactivity in patients with psoriasis. Some previous researchers have examined this subject [7-9,13,14,23], however with different methodology and never with the SSP-scale or, to our knowledge, with any other validated personality scale.

In a large Nordic psoriasis study by Zachariae et al., [8] 66% of the patients reported a subjective stress reactivity, as compared with 63% in our study. Their results suggested an indirect association between stress-reactivity and psychological vulnerability, since stress reactivity was significantly associated with more frequent use of tranquillisers, anti-depressants and tobacco, as compared with non-stress reactors.

In a study by O’Leary et al. [13] 61% of the sample reported a strong belief in stress/psychological attributes as a causal factor in their psoriasis. Consistent with our results, this belief was significantly associated with poorer levels of psychological well-being, in terms of higher levels of anxiety, depression and also with more perceived stress. Perceived stress was measured by a questionnaire and may correspond to some of the questions measuring SSP-stress susceptibility this study [13]. In accordance with our study and the O’Leary study, Fortune et al. [23] found that patients with psoriasis and a strong belief in an emotional cause of their psoriasis were more likely to experience pathological worry than those who believed the cause to be physical.

Gupta et al. [9] showed that approximately 50% of 127 patients with psoriasis reported high stress-reactivity (≥7 on a 10-point scale). High stress reactors were more likely to report difficulties with the assertion of anger. Interestingly, in our study, traits of verbal and physical aggressiveness were the only personality traits where SRs showed lower mean scores than NSRs. This may indicate that the ability to express anger is a resource in coping with stress. Furthermore, Gupta et al. [9] found that high stress reactors more often had a tendency to rely upon the approval of others. This is similar to our findings of higher scores of SSP-lack of assertiveness in patients with stress reactivity. Submissiveness and wanting approval of others is likely to create more daily stress, since social support and a sense of coherence are important factors in stress management [32,33]. In a longitudinal stydy, Kupfer and colleagues [34] found that patients with a low sense of coherence experienced their first psoriasis relapse 3.5 months after completion of treatment, whereas patients with a high sense of coherence experienced their first relapse after 10 months.

In a study of 50 patients with psoriasis, Devrimci-Ozguven et al. [14] did not find any significant differences between stress reactors and non-stress reactors regarding psychological morbidity in terms of Beck Depression Inventory and Spielberger state and trait anxiety scores. Due to a relatively small sample size, their results may be interpreted with some caution.

Patients defined as “non-stress reactors” (NSRs) in our study reported lower scores of both state and trait anxiety compared with a normative sample. Furthermore, they showed lower scores of SSP-somatic trait anxiety, SSP-psychic trait anxiety, SSP-stress-susceptibility, SSP-lack of assertiveness and SSP-mistrust. NSRs thus seem to be psychologically stable individuals, and may probably tolerate and overcome the effects of stressors in many situations. In clinical practice, it is always important to be attentive to psychological morbidity of the patients; however, these results indicate that clinicians may be less concerned about psychological morbidity in patients who do not associate their disease with stress.

Previous researchers have found stress reactivity to be associated with younger age, more psoriasis-related distress [9], greater disease severity and poorer disease-related quality of life [8], compared with non-stress reactors. However, in our study we found no significant differences between SRs and NSRs regarding disease severity, psoriasis related distress or any of the other clinical or socio-demographic variables shown in Table 1.

### Onset and stress

Nearly 50% of the patients in our study experienced that onset of their psoriasis was related to a particular stressful period or situation in life. Our results differ slightly from Zachariae’s study [8], where 35% of the subjects reported that the onset of their psoriasis occurred during a time of worry and stress. Due to the potential risk of retrospective recall bias, these results should be interpreted with caution. However, it is interesting that also patients with an experienced association between onset and stress reported significantly higher scores of both depression and traits of anxiety, compared with those without this association.

Subjective reporting and retrospective studies will always involve some degree of scientific uncertainty regarding the potential influence of recall and cognitive bias. Despite the lack of strong aetiological evidence for the association between psychological distress and psoriasis, a substantial portion of the patients in this, and previous studies [8-13], perceived psychological distress to be a causative factor in the manifestation of their disease. Patients with a chronic condition are likely to construct their own personal perceptions and ideas about their disease, in an attempt to better deal with it [35]. The beliefs that patients have about the causes of symptoms or diseases can have a profound effect on clinical management, compliance with treatment and prognosis [36]. The use of a single question for stress reactivity would provide a simple method in clinical practice to identify potentially psychologically vulnerable individuals. Results from this and previous studies [7-9,13,23] suggest important opportunities for clinicians to identify those patients who might benefit from a deeper psychological exploration.

PASI scores were estimated in the latter half of our study sample (48%), which is a limitation. However, all patients were interviewed during the same time of year i.e. early autumn, which might implicate relatively similar levels of disease severity in the entire sample. The great majority (77%) of the 48 patients scored had PASI scores representing mild disease; hence this variable was not used in the logistic regression analysis, which may be a limitation. However, PASI has often not been significantly associated with psychological morbidity in previous studies [37-39].

The methodological strengths of this study are the high participation rate and that all patients were interviewed by the same researcher. All patients were consecutively recruited from the same clinic, and only eight of 109 patients declined participation. Moreover, the total study sample showed a homogeneous personality profile and was not more anxious or depressed than the general population [24,25]. Thus, it may be assumed that the patients in this study represent a psychiatrically normal sample in further analyses of stress reactivity and interpretation of results.

## Conclusions

According to our results, patients who perceive stress as a causal factor for exacerbation of their disease seem to have a more vulnerable psychological constitution. This finding suggests important opportunities for clinicians to identify patients who might benefit from additional psychological exploration and support.
